# Simultaneous determination of major type A and B trichothecenes, zearalenone and certain modified metabolites in Finnish cereal grains with a novel liquid chromatography-tandem mass spectrometric method

**DOI:** 10.1007/s00216-015-8676-4

**Published:** 2015-05-03

**Authors:** Alexis V. Nathanail, Jenna Syvähuoko, Alexandra Malachová, Marika Jestoi, Elisabeth Varga, Herbert Michlmayr, Gerhard Adam, Elina Sieviläinen, Franz Berthiller, Kimmo Peltonen

**Affiliations:** Chemistry and Toxicology Unit, Research and Laboratory Department, Finnish Food Safety Authority (Evira), Mustialankatu 3, 00790 Helsinki, Finland; Christian Doppler Laboratory for Mycotoxin Metabolism and Center for Analytical Chemistry, Department for Agrobiotechnology (IFA-Tulln), University of Natural Resources and Life Sciences, Vienna (BOKU), Konrad Lorenz Str. 20, 3430 Tulln, Austria; Product Safety Unit, Control Department, Finnish Food Safety Authority (Evira), Mustialankatu 3, 00790 Helsinki, Finland; Department of Applied Genetics and Cell Biology, University of Natural Resources and Life Sciences, Vienna (BOKU), Konrad Lorenz Str. 24, 3430 Tulln, Austria; Plant Analysis Unit, Research and Laboratory Department, Finnish Food Safety Authority (Evira), Mustialankatu 3, 00790 Helsinki, Finland; Finnish Safety and Chemicals Agency (Tukes), Opastinsilta 12, 00521 Helsinki, Finland

**Keywords:** *Fusarium* mycotoxins, Masked mycotoxins, LC-MS/MS, Method validation, Survey

## Abstract

A reliable and sensitive liquid chromatography-tandem mass spectrometric method was developed for the simultaneous quantitative determination in cereals of the *Fusarium* mycotoxins HT-2 toxin, T-2 toxin, deoxynivalenol, nivalenol and zearalenone, as well as the modified metabolites 3-acetyl-deoxynivalenol, α-zearalenol, β-zearalenol, deoxynivalenol-3-glucoside, HT-2-3-glucoside, nivalenol-3-glucoside, zearalenone-14-glucoside, zearalenone-14-sulphate, zearalenone-16-glucoside, α-zearalenol-14-glucoside and β-zearalenol-14-glucoside. The ‘dilute and shoot’ approach was used for sample preparation after extraction with acetonitrile:water:acetic acid (79:20:1, *v*/*v*/*v*). Separation was carried out using reversed-phase liquid chromatography, and detection was performed using tandem mass spectrometry in the selected reaction monitoring mode. The method was in-house validated according to performance characteristics, established in Commission Regulation EC No 401/2006 and Commission Decision EC No 657/2002, prior to its application in a nationwide survey for the analysis of barley, oat and wheat samples (*n* = 95) harvested in Finland during 2013. Deoxynivalenol and its glucosylated form were the most abundant of the analytes, being detected in 93 and 81 % of the samples, respectively. Concentrations of deoxynivalenol were unusually high in 2013, especially in oats, with some cases exceeding the maximum legislative limits for unprocessed oats placed on the market for first-stage processing. All modified mycotoxins analysed were detected, and the natural occurrence of some of these compounds (e.g. zearalenone-16-glucoside and nivalenol-3-glucoside) in barley, oats and/or wheat was documented for the first time.

**Electronic supplementary material:**

The online version of this article (doi:10.1007/s00216-015-8676-4) contains supplementary material, which is available to authorized users.

## Introduction

Plant pathogenic species of the genus *Fusarium* are widespread pathogens of small-grain cereals, capable of producing an array of heterogeneous mycotoxins. These fungal secondary metabolites exert a broad range of biological activities that may cause acute or chronic health problems in humans and animals after the consumption of mycotoxin-contaminated commodities. In northern Europe, the most important fusariotoxins in cereal grains are trichothecenes and zearalenone (ZEN) [1].

**Fig. 1:**
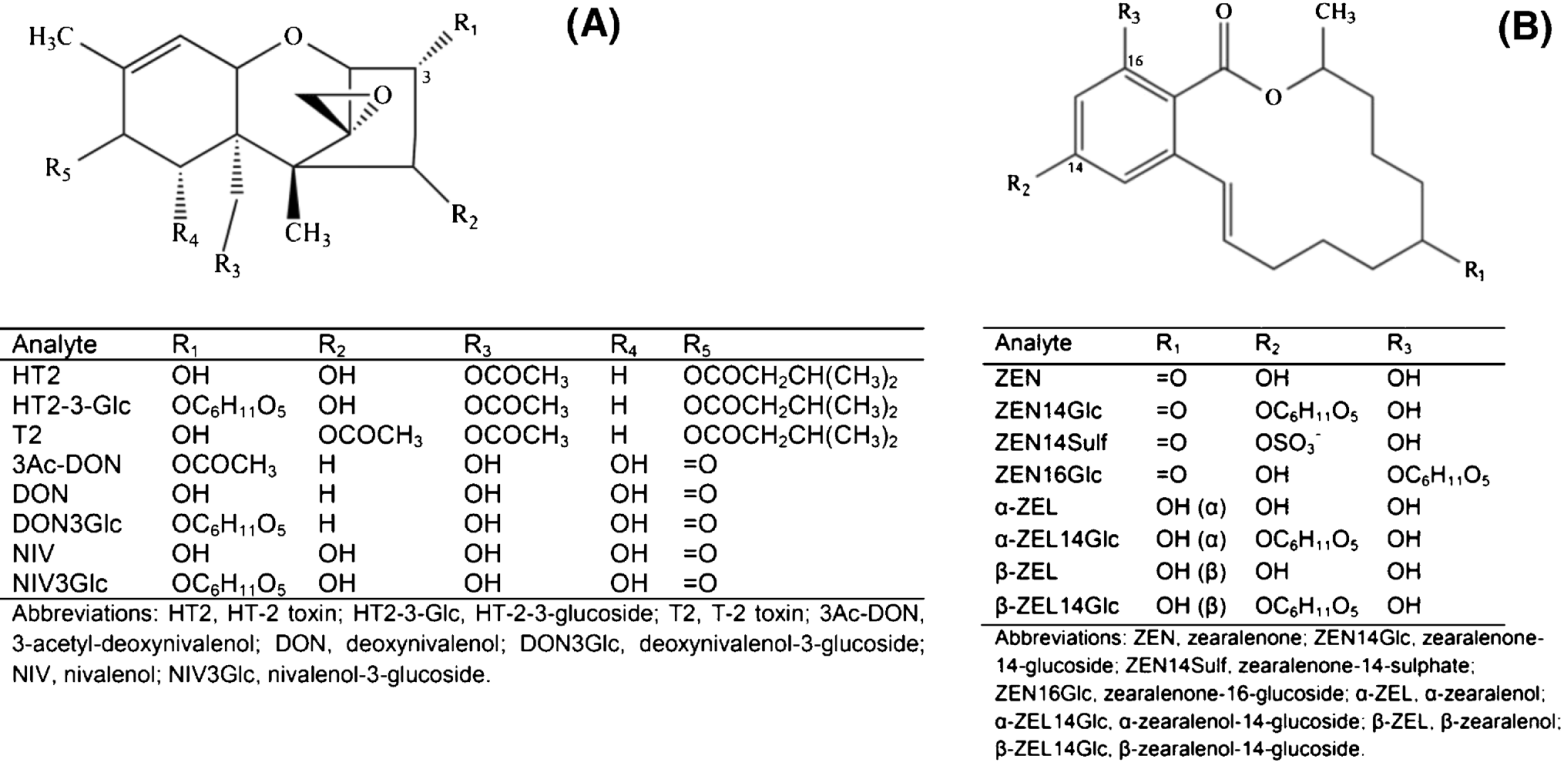
Chemical structures of native and modified type A and B trichothecenes (**A**) and zearalenone (**B**)

To date, more than 200 trichothecenes have been identified [2] and are classified according to their structure into four types: A, B, C and D [3]. These compounds share a common cyclic sesquiterpene skeleton with a C-12, C-13 epoxy ring (Fig. 1A). Manifestations of toxic effects following exposure to trichothecenes include haematotoxicity, neurotoxicity and immunosuppression, causing animal feed refusal, growth retardation and vomiting [4, 5]. Toxins belonging to the type A group, such as HT-2 toxin (HT2) and T-2 toxin (T2), as well as the type B trichothecenes deoxynivalenol (DON) and nivalenol (NIV) are relevant to food safety due to their occurrence in edible crops. According to survey data from Finland and other Nordic countries, oats are usually more severely affected by high DON and HT2/T2 concentrations compared to barley and wheat [6]. During the past decade, high incidences of HT2 and T2 have been documented in raw oats from several northern European countries, with the mean concentrations varying considerably from year to year (e.g. [7, 8]). For NIV, a negative relationship with the abundance of high DON levels has been observed, which can mainly be attributed to the production of these mycotoxins by different *Fusarium* species [9].

ZEN is a macrocyclic β-resorcyclic acid lactone consisting of a ring with two ketones and an aromatic ring substituted with two hydroxyl groups (Fig. 1B). ZEN and its phase I metabolites, α-zearalenol (α-ZEL) and β-zearalenol (β-ZEL), exert strong oestrogenic effects on vertebrates by binding to the oestrogen receptors ER-α and ER-β [10]. Subchronic and long-term toxicity experiments have confirmed the oestrogenic activity of ZEN in livestock, causing infertility, reduced foetal weight or embryonic death [11]. Oats have been found to contain the highest ZEN concentrations in Finland (up to 1690 μg/kg) according to survey data obtained during the period 1999–2009 [9].

Trichothecenes and ZEN, similarly to other xenobiotics, are metabolised in living organisms by natural detoxification mechanisms into products distinct from the native toxins. These compounds may be activated versions of the native form as a result of phase I metabolism, or phase II conjugated metabolites with glucose, sulphate or amino acid moieties, and are defined as ‘modified mycotoxins’ [12]. The term ‘masked mycotoxins’, which was originally used to describe altered forms of mycotoxins, henceforth refers exclusively to plant-generated mycotoxin metabolites [13]. Compounds formed during food manufacturing processes or metabolites excreted directly by fungi (e.g. 3-acetyl-deoxynivalenol, 3Ac-DON) are also classified as modified mycotoxins. Several *in planta* trichothecene conjugates have already been reported, including deoxynivalenol-3-glucoside (DON3Glc) [14], HT-2-3-glucoside (HT2-3-Glc) [15] and nivalenol-3-glucoside (NIV3Glc) [16]. In addition to the major ZEN metabolites, the ZELs, other plant-specific metabolites, have been described, such as zearalenone-14-glucoside (ZEN14Glc), α-ZEL-14-glucoside (α-ZEL14Glc), β-ZEL-14-glucoside (β-ZEL14Glc), ZEN-14-sulphate (ZEN14Sulf) [17] and the recently reported zearalenone-16-glucoside (ZEN16Glc) [18]. From a toxicological perspective, DON3Glc and ZEN14Glc are the most studied plant-generated modified mycotoxins, and according to current knowledge, their inherent toxicity is lower than that of their respective native forms. However, toxicological implications may arise from the reactivation of these compounds by the action of intestinal microbiota during mammalian digestion [19, 20].

In order to conduct a comprehensive exposure assessment concerning modified mycotoxins and investigate the multi-component nature of mycotoxin-contaminated samples, improved analytical methodologies are necessary. The majority of modern analytics for mycotoxin quantification in cereals and cereal-derived foodstuffs rely on liquid chromatography-tandem mass spectrometry (LC-MS/MS) [21]. LC-MS-based methods can render multiple native mycotoxins and potentially also conjugated forms directly accessible for detection [22]. However, methods that have initially been developed for determination of parent mycotoxins may suffer from inherent problems (e.g. poor recovery of polar analytes), chromatographic deficiencies or inadequate sensitivity when used for analysis of modified mycotoxins [23]. During the past few years, dedicated multi-analyte LC-MS/MS methods have been developed for modified mycotoxins [24, 25], although these are not applicable in the majority of laboratories due to the lack of analytical standards required for their accurate measurement. Such methods, as with most multi-mycotoxin methods, have been based on the ‘dilute and shoot’ approach, since no clean-up has been found suitable to accommodate the wide range of analyte polarities. Recently, on-line clean-up was successfully applied for DON and DON3Glc determination, overcoming limitations related to the dilute and shoot approach, such as severe matrix effects and low sensitivity [26].

In 2006, the European Commission (EC) set maximum levels (MLs) for 11 mycotoxins, including DON and ZEN, in certain foods intended for human consumption (Commission Regulation EC No 1881/2006 [27]). Regarding T2 and HT2, so-called indicative levels for monitoring the sum of these *Fusarium* mycotoxins in cereals and cereal-derived products were published in Commission Recommendation 2013/165/EU [28]. The same document also highlights the need for data on the occurrence of conjugated mycotoxins. The European Food Safety Authority (EFSA) recently published a scientific opinion on the risks to human and animal health related to the presence of modified forms in food and feed, addressing the need for properly validated and sensitive analytical methods [29]. In this context, the aim of our study was to develop a reliable and sensitive LC-MS/MS method for the determination of T2, HT2, DON, NIV and ZEN, as well as several of their modified metabolites in cereal grains. The method was in-house validated for barley, oats and wheat according to available legislative method performance criteria [30, 31] and was used to examine the occurrence of native and modified mycotoxins on Finnish grain samples.

## Materials and methods

### Reagents and standards

HPLC-grade acetonitrile, methanol, acetic acid and hexane were purchased from J.T. Baker (Deventer, the Netherlands). Ammonium formate (MS grade) and formic acid (p.a. grade) were obtained from Sigma-Aldrich (Steinheim, Germany), whereas ammonium acetate (MS grade) was purchased from VWR International (Leuven, Belgium). Water was purified with a Milli-Q Plus system (Millipore, Espoo, Finland). Analytical standards for HT2, T2, 3Ac-DON, DON, NIV, ZEN, α-ZEL and β-ZEL were obtained from Sigma-Aldrich in solid form. The modified mycotoxins DON3Glc [32], ZEN14Glc, α-ZEL14Glc, β-ZEL14Glc [33] and ZEN16Glc [18], as well as ZEN14Sulf [34], HT2-3-Glc and NIV3Glc (Michlmayr et al., in preparation), were produced by us at the University of Natural Resources and Life Sciences, Vienna (BOKU). All native mycotoxins used for the production of modified forms were purchased as analytical standards in solid form from Romer Labs (Tulln, Austria). All production procedures ended with isolation of the modified toxins by preparative HPLC under reversed-phase conditions. Their identity and purity (>98 %) were verified by NMR and LC-UV measurements. Individual stock solutions for all analytes were prepared by dissolving the solid substance in acetonitrile. In total, eight working solutions were prepared on a weekly basis by diluting the stock solutions to the appropriate concentrations. The solutions were stored at −20 °C and were brought to room temperature in the dark before use.

### Survey samples

**Fig. 2:**
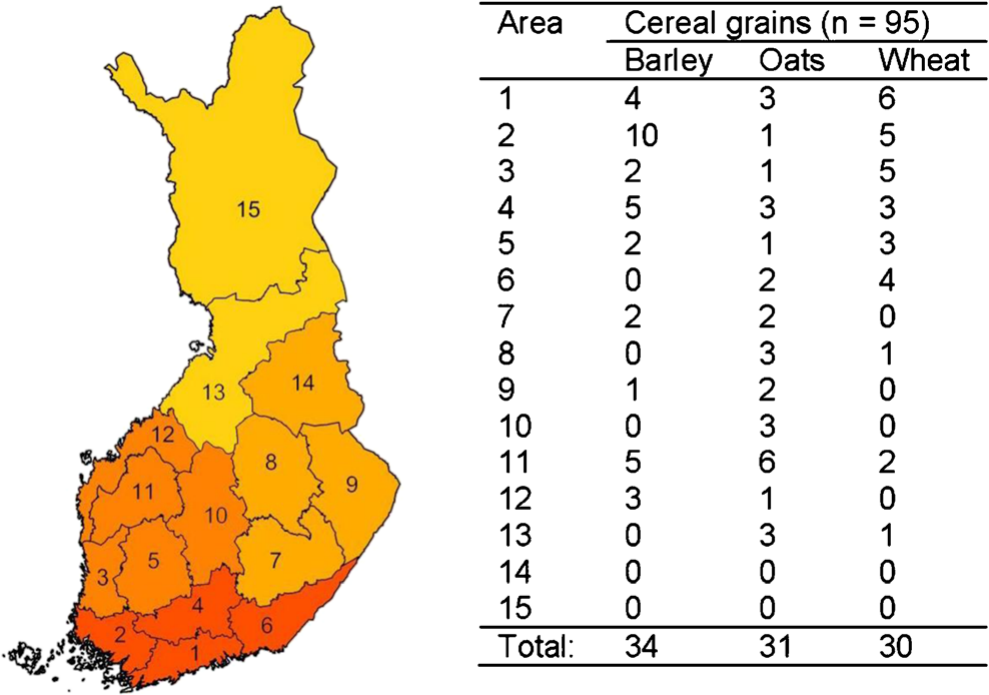
Origin of the cereal samples analysed during the survey study. The *colours* represent the different regions: North, East, South and West

Spring wheat, oat and barley samples from the quality monitoring programme of the Finnish grain harvest were analysed for *Fusarium* mycotoxins and their *in planta* metabolites. The quality monitoring programme is based on grain samples sent in by farmers. The survey samples in this study (*n* = 95) represented commonly cultivated small-grain cereal varieties in Finland from different geographic areas and were randomly chosen from a total of 1117 samples of the 2013 harvest (Fig. 2). Areas 14 and 15 are not represented in this survey, since no significant cereal production takes place there. According to the background information provided by farmers, 77 % of the spring wheat samples were intended for the food industry, 13 % for feed and 10 % for seed. Half of the barley samples were malting varieties, 38 % were feed varieties and 12 % were cultivated for other purposes. Concerning oats, 16 % were intended for food and 84 % for feed, farm trade and seed.

The samples analysed during the survey consisted of several subsamples collected from arbitrary points in each field (2 kg per sample). After harvest, the samples were air-dried to a moisture content below 15 % to avoid fungal growth. Prior to analysis, each 2-kg sample was finely ground in its entirety using a Retsch ZM 200 grinder (Retsch GmbH, Haan, Germany) and stored at room temperature in plastic containers.

The growing period of 2013 was warm in Finland, with the average mean daily temperatures being 1.0–2.0 °C above the long-term average (17.6 °C) throughout the country. The spring sowing started with dry weather conditions, but during the growing season, there were regional variations in rainfall levels. Precipitation was less than a third of the long-term average of 190 mm in the southwestern parts of Finland, in contrast to the eastern parts, where it was generally higher than the long-term average (>250 mm). During the harvest time, the weather was generally dry. The meteorological data of the study were obtained from the Internet services of the Finnish Meteorological Institute (http://www.fmi.fi).

### Spiking experiments and sample preparation

For the wheat and barley spiking experiments, commercially available flours were used as a blank matrix. In the case of oats, oat kernels received from the Finnish grain quality monitoring programme were used. The kernels were finely ground with a Retsch ZM 200 grinder and analysed for use as blank material by verifying that no or only trace levels of target analytes were detected. Sample preparation was performed according to a slightly adjusted dilute and shoot procedure as described by Sulyok et al. [35]. Briefly, 0.500 ± 0.005 g of cereal flour was weighed into glass extraction tubes with screw caps. For validation, the blank cereal samples were then spiked with the appropriate aliquot of the final multi-analyte working solution prior to extraction. The spiking levels (Table 1) were chosen to cover available mycotoxin/modified mycotoxin occurrence data, as well as the legislative limits set for mycotoxins [27, 28]. The samples were allowed to soak overnight in the dark at room temperature. The extraction was performed by adding 2 mL extraction solution (acetonitrile:water:acetic acid, 79:20:1, *v*/*v*/*v*), followed by vortexing (30 s) and mixing in a VKS-75 Control horizontal shaker (Edmund Bühler, Bodelshausen, Germany) at 200 rpm for 90 min. After extraction, the samples were centrifuged at 2700×*g* for 10 min with a Heraeus Multifuge S-R centrifuge (Hanau, Germany). Finally, 350 μL of the supernatant of each sample was transferred into HPLC vials and diluted with an equal amount of dilution solvent (acetonitrile:water:acetic acid, 20:79:1, *v*/*v*/*v*) and injected into the LC-MS/MS system. For oats, an additional defatting step was employed, where 1 mL of hexane was added to the extract (ca. 1.2 mL) and the mixture was shaken for 10 min, followed by centrifugation at 2700×*g* for 5 min. The hexane layer was removed with a Pasteur pipette, and 350 μL of the extract layer was diluted with 350 μL dilution solvent and transferred into HPLC vials for analysis. The survey samples were prepared in duplicate using the same process as for the validation samples.

**Table 1:** Spiked target analyte concentration levels in barley, oats and wheat blank matrices during validation of the LC-MS/MS method

Analyte	Spiking level (μg/kg)		
	Low	Medium	High
3Ac-DON	20	80	160
DON	100	400	800
DON3Glc	25	100	200
HT2	10	50	100
HT2-3-Glc	8	30	60
NIV	40	80	160
NIV3Glc	20	40	80
T2	10	50	100
ZEN	15	60	120
ZEN14Glc	8	30	60
ZEN14Sulf	8	30	60
ZEN16Glc	8	30	60
α-ZEL	8	30	60
α-ZEL14Glc	8	30	60
β-ZEL	8	30	60
β-ZEL14Glc	8	30	60

### LC-MS/MS analysis

The samples were analysed using a Waters Acquity™ UPLC system (Waters Co., Milford, MA, USA) coupled to a Waters Xevo™ triple quadrupole (TQ) MS (Micromass, Manchester, UK), equipped with an electrospray ionisation (ESI) interface. A Waters Atlantis^®^ T3 column (150 × 3.0 mm, 3.0 μm) with a matching Atlantis T3 pre-column was used for chromatographic separation, which was carried out at room temperature with a flow rate of 0.35 mL/min using binary gradient elution. Eluent A was water buffered with 10 mM ammonium acetate, whereas eluent B was acetonitrile. The gradient programme was initiated with a holding time of 2 min at 5 % B, after which 55 % B was linearly reached within 5 min. These conditions were maintained for 2 min before a further linear increase of B to 90 % within 6 min and isocratic elution for 2 min. Finally, the starting conditions were switched back (at 17 min) followed by a 4-min hold time to allow re-equilibration, resulting in a total run time of 21 min. The injection volume was 5 μL and the autosampler was maintained at 10 °C.

The ESI source parameters were as follows: capillary voltage, 2.9 kV; source temperature, 150 °C; desolvation temperature, 350 °C and desolvation gas flow, 950 L/h. Precursor and product ion selection and optimisation of the MS/MS conditions (e.g. cone voltages and collision energies) were conducted using the Waters IntelliStart™ software by infusion (10 μL/min) of individual analyte standards (0.5–1.5 μg/mL) dissolved in acetonitrile. The MS detection was performed with the selected reaction monitoring (SRM) mode, resulting in automatically optimised dwell times of 22–151 ms. Two SRM transitions were monitored for each compound; the one with the highest intensity was selected as the quantifier ion and the other as the qualifier. The cone voltages and collision energies are presented in Table 2, along with other parameters of the analytes included in this method. Data acquisition and processing were performed with Waters MassLynx™ v.4.1 and QuanLynx™ v.4.1 software.

**Table 2:** Precursor/product ion pairs and LC-MS/MS parameters in the ESI-SRM mode for detection of the measured analytes (sorted by retention times)

Analyte	Retention time (min)	Molecular ion	Precursor ion (*m*/*z*)	Product ions (*m*/*z*)	Cone voltage (V)	Collision energy (eV)
				Quantifier, qualifier		
NIV3Glc	6.62	[M+CH_3_COO]^−^	533.3	473.3, 263.0	24	12, 24
NIV	6.76	[M+CH_3_COO]^−^	371.2	311.0, 281.1	20	10, 12
DON3Glc	6.93	[M+CH_3_COO]^−^	517.2	457.1, 427.1	26	14, 20
DON	7.38	[M+H]^+^	297.3	249.1, 91.0	18	10, 36
β-ZEL14Glc	8.36	[M−H]^−^	481.1	319.1, 275.1	26	14, 34
ZEN16Glc	8.59	[M+NH_4_]^+^	498.4	319.1, 283.2	16	14, 28
α-ZEL14Glc	8.72	[M−H]^−^	481.3	293.2, 301.2	50	30, 34
HT2-3-Glc	8.95	[M+NH_4_]^+^	604.4	157.0, 105.0	20	36, 68
3Ac-DON	8.98	[M−H]^−^	337.0	173.0, 307.1	26	6, 14
ZEN14Glc	9.22	[M+NH_4_]^+^	498.4	319.1, 283.2	16	14, 28
ZEN14Sulf	9.59	[M−H]^−^	397.0	317.1, 175.0	32	22, 34
HT2	10.44	[M+NH_4_]^+^	442.4	263.1, 215.1	12	10, 12
β-ZEL	11.59	[M−H]^−^	319.1	174.1, 159.8	44	28, 34
α-ZEL	12.76	[M−H]^−^	319.1	159.8, 130.0	40	28, 34
T2	13.29	[M+NH_4_]^+^	484.4	305.1, 185.1	18	14, 22
ZEN	14.58	[M−H]^−^	317.1	174.9, 131.0	40	24, 28

### Method validation and data evaluation

The method was validated for wheat, barley and oats in terms of linearity, specificity, apparent recovery, repeatability, inter-day precision, the limit of detection (LOD) and the limit of quantification (LOQ), following the performance criteria and recommendations of Commission Regulation EC No 401/2006 [30] and Commission Decision EC No 657/2002 [31]. Each of the three concentration levels for validation included six spiked samples for each matrix. Blank barley, oat and wheat samples (*n* = 20 per matrix) were also analysed along with the spiked ones. The process was repeated on 3 days. Prior to validation, the efficiency of different extraction solutions was investigated. Furthermore, matrix effects were estimated as the signal suppression and enhancement (SSE) ratio to determine whether the use of matrix-assisted calibration curves in the experiments was necessary. The SSE values were obtained in triplicate as the percentage of the slope of calibration curves prepared in spiked extracts divided by the slope of curves prepared in neat solution [35].

The MS response (peak area) was plotted against the analyte concentration to calculate a linear, 1/*x* weighted calibration curve. The concentration ranges of the five-point calibration curves were 20–1200 μg/kg for DON and between 10 and 250 μg/kg for 3Ac-DON, DON3Glc, HT2, NIV, NIV3Glc, T2 and ZEN, with the lowest calibration point based on the LOQ values of each analyte. For α- and β-ZEL, as well as for all other modified forms, calibration ranges of 5–100 μg/kg were used. Linearity was evaluated on neat standard and spiked matrix curves by fitting the data with a linear regression model. The LODs were determined by 20 blank samples per matrix, as the sum of the mean analyte signal and three times the standard deviation (SD). The concentration values for LOD were calculated by comparing the areas with the corresponding concentrations of the lowest spiked levels of the respective calibrant. The LOQ is presented as three times the LOD. Specificity was estimated as the maximum permitted tolerances of relative quantifier/qualifier ion ratios of each target analyte and compared to those obtained in spiked matrix. Moreover, the retention time of the analyte had to match that obtained by the neat standard within a margin of ±2.5 %. To calculate the apparent recovery, spiked sample concentrations were measured using the matrix-assisted calibration curves as the percentage of the measured analyte concentration divided by the spiked concentration. Repeatability (RSD_r_), expressed as relative standard deviation, was determined as the variation within daily results. Similarly, inter-day precision (RSD_R_) was assessed as the day-to-day variation. Validation data were processed with Microsoft Excel^®^ 2010 (Microsoft Co., Redmond, WA, USA). The reported survey sample concentrations were calculated as the mean value of two replicates. For the calculation of the contamination on average, concentrations below the respective LOQ were assigned a value of LOQ/2, whereas values below LOD were treated as non-contaminated.

## Results and discussion

### Method development and optimisation

**Fig. 3:**
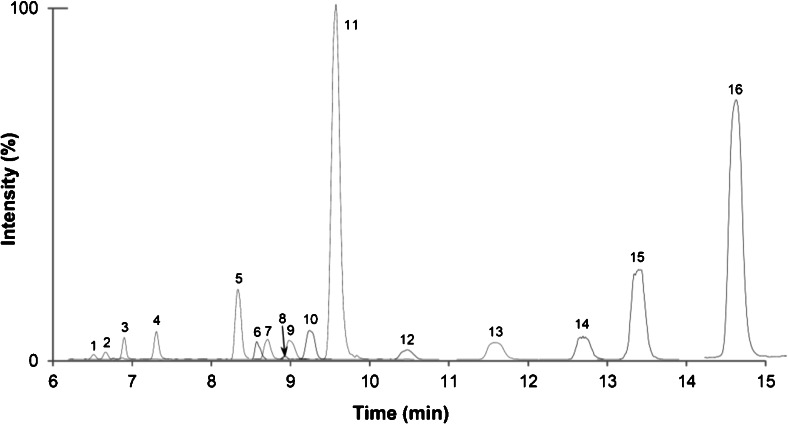
Overlayed quantifier extracted ion chromatograms of a blank wheat sample spiked at the medium level, with *1* NIV3Glc; *2* NIV; *3* DON3Glc; *4* DON; *5* β-ZEL14Glc; *6* ZEN16Glc; *7* α-ZEL14Glc; *8* HT2-3-Glc; *9* 3Ac-DON; *10* ZEN14Glc; *11* ZEN14Sulf; *12* HT2; *13* β-ZEL; *14* α-ZEL; *15* T2; *16* ZEN

The method development was initiated by infusing individual analyte solutions into the MS, operating in both positive and negative ESI scan modes. ZEN and its derivatives, with the exception of ZEN14Glc and ZEN16Glc, as well as most of the type B trichothecene-based analytes showed higher intensities in the negative ESI mode, whereas type A trichothecene-based derivatives were more easily ionised in the positive mode. In order to achieve the best sensitivity for all analytes in the MS using only a single LC run, proper chromatographic separation and frequent polarity switching of the interface during each run was required (Table 2). Initially, a Waters reversed-phase C18 column was tested, but the retention of the more polar modified mycotoxins (e.g. NIV3Glc and DON3Glc) was not satisfactory. Gradient elution adjustments only slightly improved the poor retention of some polar analytes. With the use of the Atlantis column, which is recommended by the manufacturer for the separation of compounds with diverse polarities, significantly better retention for the polar analytes was accomplished and also baseline separation between most isomeric pairs. However, the isomeric compounds ZEN14Glc/ZEN16Glc co-eluted, making them indistinguishable, as they share common transitions. For ZEN14Glc and ZEN16Glc, a short isocratic elution phase between 7 and 9 min had to be introduced during the linear gradient programme to achieve chromatographic separation. The retention times of the 16 analytes ranged from 6.62 min (NIV3Glc) to 14.58 min (ZEN) and are presented in Fig. 3 in the form of overlayed extracted ion chromatograms.

Before finalising the method, different extraction solutions were assessed for their efficiency in extracting the target analytes. All native mycotoxins together with the modified mycotoxins 3Ac-DON, DON3Glc, α-ZEL and β-ZEL were included in the tests. In these trials, four extraction mixtures were used: (i) acetonitrile:water:acetic acid (79:20:1, *v*/*v*/*v*), (ii) acetonitrile:water:formic acid (79:20.9:0.1, *v*/*v*/*v*), (iii) acetonitrile:water (84:16, *v*/*v*) and (iv) methanol:water (84:16, *v*/*v*). During the extraction process, 500 μg/kg of each target analyte was spiked in three replicates for barley and wheat matrices; the process was repeated twice. Our tests verified the applicability of the extraction solution (i), which has been successfully used in multi-mycotoxin methods based on the dilute and shoot approach for type A and B trichothecenes, ZEN and some of their modified forms [24, 25, 36]. In particular, for the native mycotoxins, overall extraction recoveries of more than 90 % were achieved with SD values ≤10 %. In the case of modified mycotoxins, recoveries with (i) were generally higher compared to those of the other mixtures, except for β-ZEL, which was extracted at around 80 % (data not shown). The extraction solution (ii) performed better for HT2 (96 %) and NIV (93 %) compared to 90 and 91 % obtained with (i), respectively. Overall, (i) and (ii) yielded similar results, higher than (iii) and (iv) especially for the more polar analytes, but (i) was preferable in matching the chromatographic conditions. Concerning matrix effects, in the three cereal matrices, no severe signal suppression to values more than half of the neat standard was observed for any of the analytes (Table 3 for wheat and in the Electronic Supplementary Material (ESM) Tables S1 and S2 for barley and oats). On the other hand, significant matrix-induced enhancement was observed for 3Ac-DON in all three matrices (109–123 %) and HT2-3-Glc (121 %) in barley. In general, ZEN metabolites were found to have a fluctuating matrix-related behaviour among the different cereals. Therefore, matrix-assisted calibration spiked prior to extraction and prepared identically to the samples was used for quantification of the target analytes, in order to take into account extraction recovery and matrix effects.

**Table 3:** Method performance characteristics and validation parameters determined in wheat

Analyte	SSE (%)	LOD (μg/kg)	LOQ (μg/kg)	*R* ^2^	Ion ratio (Quan/Qual)	Apparent recovery ± RSD_R_ (%) (*n* = 18)		
						Low	Medium	High
3Ac-DON	123	3.7	11.1	0.991	0.3	97 ± 34	96 ± 9	100 ± 10
DON	80	1.3	3.9	0.988	1.6	98 ± 18	97 ± 12	99 ± 8
DON3Glc	76	2.2	6.6	0.998	1.1	99 ± 10	96 ± 7	100 ± 5
HT2	101	3.0	9.0	0.997	1.0	107 ± 17	99 ± 11	101 ± 9
HT2-3-Glc	95	3.6	10.8	0.991	0.6	108 ± 26	97 ± 12	102 ± 6
NIV	67	3.2	9.6	0.986	1.4	95 ± 16	93 ± 8	95 ± 4
NIV3Glc	75	4.3	12.9	0.985	4.4	100 ± 21	93 ± 16	100 ± 8
T2	101	1.4	4.2	1.000	0.8	101 ± 8	99 ± 4	101 ± 4
ZEN	82	1.9	5.7	0.998	1.3	99 ± 14	98 ± 4	99 ± 4
ZEN14Glc	92	0.1	0.3	0.997	2.5	111 ± 11	96 ± 8	111 ± 6
ZEN14Sulf	75	0.2	0.6	0.997	7.4	100 ± 13	94 ± 5	100 ± 5
ZEN16Glc	68	0.3	0.9	0.988	3.1	112 ± 19	101 ± 8	112 ± 8
α-ZEL	87	0.1	0.3	0.998	1.1	96 ± 13	95 ± 7	96 ± 4
α-ZEL14Glc	92	0.5	1.5	0.994	2.3	94 ± 19	95 ± 10	94 ± 8
β-ZEL	84	0.3	0.9	0.997	1.1	101 ± 12	94 ± 6	101 ± 5
β-ZEL14Glc	86	0.2	0.6	0.997	8.7	103 ± 23	96 ± 25	103 ± 4

*SSE* signal suppression/enhancement ratio, *LOD* limit of detection, *LOQ* limit of quantification, *R* ^*2*^ coefficient of determination, *Quan* quantifier ion, *Qual* qualifier ion, *RSD*_*R*_ inter-day precision

### Method performance characteristics

The developed LC-MS/MS method was successfully validated for the determination of 16 analytes (3Ac-DON, DON, DON3Glc, HT2, HT2-3-Glc, NIV, NIV3Glc, T2, ZEN, ZEN14Glc, ZEN14Sulf, ZEN16Glc, α-ZEL, α-ZEL14Glc, β-ZEL and β-ZEL14Glc) in barley, oats and wheat. The method performance characteristics obtained during the validation procedure are presented in Table 3 for wheat and in ESM Tables S1 and S2 for barley and oats, respectively.

LOD values ranged from 0.1 to 4.1 μg/kg in barley, 0.1 to 5.3 μg/kg in oats and 0.1 to 4.3 μg/kg in wheat, allowing the detection of mycotoxins and their modified forms in the low microgram-per-kilogram region. In general, DON, T2 and the derivatives of ZEN had the lowest LODs, and subsequently LOQ values, in all matrices. Among the trichothecene derivatives, the highest LODs were observed for 3Ac-DON, HT2-3-Glc and NIV3Glc. Calibration curves were linear over the respective working range of each analyte, with the coefficient of determination (*R*^2^) values being 0.982–1.000 for the five-point matrix-assisted calibration curves. For those survey samples whose concentrations exceeded the linear range of analytes, appropriate dilutions with blank extracts were performed. Specificity of the target analytes with SRM was confirmed by the presence of analyte quantifier/qualifier transitions at the correct retention times in neat standard. This parameter was also verified by the absence of any interfering peaks in the SRM channels in the 20 blank samples analysed per matrix.

Accuracy and precision were evaluated by recovery experiments. The apparent recoveries varied between 90 and 116 % in barley, 84 and 115 % in oats and 93 and 112 % in wheat for all analytes. For those analytes for which performance criteria were available, the apparent recoveries were in agreement with the recommended legislative values (typically 70–120 %). For compounds for which no such values exist, acceptable apparent recovery was also achieved. In the case of NIV in oats, two outliers were removed from the low level because of a spiking error. The matrix effects (expressed as SSE) ranged between 59 and 121 % for barley, 53 and 122 % for oats and 67 and 123 % for wheat. Based on these results, it was proven that the extraction solution (acetonitrile:water:acetic acid, 79:20:1, *v*/*v*/*v*) uniformly suited all analytes included in the method. RSD_r_ and RSD_R_ were also calculated for the three concentration levels, but only the RSD_R_ values are presented here. The RSD_r_ values were relatively high for the glucosylated NIV and HT2 derivatives but never exceeded though the 40 % limit set in EC No 401/2006 for the native HT2 at the low concentration level (100–200 μg/kg) [30]. Finally, RSD_R_ values were below the 40–60 % limit required by the same regulation for analysis of the native DON, HT2, T2 and ZEN, as no performance criteria are currently available for modified forms.

### Naturally contaminated raw cereal samples

**Fig. 4:**
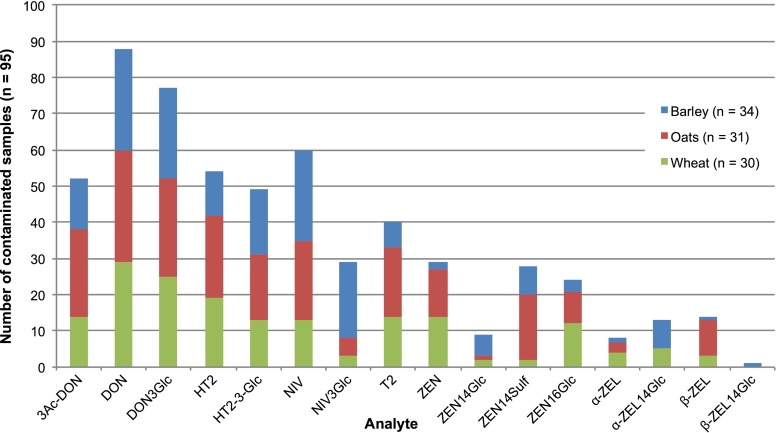
Overview of the *Fusarium* mycotoxins and modified mycotoxins detected in Finnish cereal grains in 2013 (*n* = 95)

The developed and validated LC-MS/MS method was applied for the analysis of 95 cereal grain samples from Finland harvested during 2013, and the results are summarised in Table 4. Figure 4 presents an overview of the detected mycotoxins and modified mycotoxins in barley, oats and wheat. The results of our survey revealed considerable variation between different commodities. The highest contamination of the studied mycotoxins was observed in oats, followed by wheat and barley. The most frequently detected native mycotoxin was DON (incidence 93 % of the samples analysed), followed by NIV (63 %) and HT2 (57 %). DON was also found at the highest concentrations, and particularly in wheat and oats, with 32 % of the analysed oats exceeding the legislative ML of 1750 μg/kg set for DON regarding unprocessed oats placed on the market for first-stage processing [27]. For wheat, the percentage of samples exceeding the ML (1250 μg/kg) was 23 %, whereas no barley sample exceeded the same level. 3Ac-DON (55 %) was only found at increased levels in oats, in samples also showing high concentrations of DON. One wheat and one oat sample also exceeded the ML set for ZEN (100 μg/kg), and both were also highly contaminated with DON (1660 and 23,800 μg/kg, respectively). Such DON levels are unusually high compared to those of previous years based on quantitative results from the Finnish grain quality monitoring programme (1999–2009) [9]. Only minor amounts of HT2 and T2 were detected in barley and wheat. In contrast, the prevalence of these toxins in oats was significantly more pronounced. Nonetheless, only one sample exceeded the indicative value for the sum of these toxins (1000 μg/kg) in Recommendation 2013/165/EU for unprocessed oats [28]. NIV contamination was relatively low in barley and wheat samples in comparison to the levels found in oats, with a mean concentration of 635 μg/kg that can be attributed to four very highly contaminated samples (>1400 μg/kg).

**Table 4:** Concentration levels of *Fusarium* mycotoxins and modified mycotoxins in Finnish cereal grains in 2013 (*n* = 95)

Analyte	Barley (*n* = 34)				Oats (*n* = 31)				Wheat (*n* = 30)			
	Percentage > LOD	Average (μg/kg)^a^	95th percentile (μg/kg)	Maximum (μg/kg)	Percentage > LOD	Average (μg/kg)^a^	95th percentile (μg/kg)	Maximum (μg/kg)	Percentage > LOD	Average (μg/kg)^a^	95th p ercentile (μg/kg)	Maximum (μg/kg)
3Ac-DON	41.2	<LOQ	18.3	23.6	77.4	341	1280	2720	46.7	24.6	64.9	71.0
DON	82.4	234	802	1180	100	2690	11,800	23,800	96.7	866	3710	5510
DON3Glc	73.5	148	594	1300	87.1	806	4580	6600	83.3	174	730	922
HT2	35.3	20.0	38.3	39.5	74.2	159	419	1830	63.3	<LOQ	15.3	15.9
HT2-3-Glc	52.9	<LOQ	27.1	38.5	58.1	41.4	100	300	43.3	15.0	40.3	42.6
NIV	73.5	96.6	262	302	71.0	635	2440	4940	43.3	48.9	73.0	74.0
NIV3Glc	61.8	25.2	53.8	65.3	16.1	36.9	57.8	58.3	10.0	23.1	32.2	33.6
T2	20.6	10.7	16.9	18.1	61.3	60.1	242	548	46.7	<LOQ	4.9	5.4
ZEN	5.9	13.7	16.6	17.0	41.9	76.9	326	675	46.7	37.7	130	234
ZEN14Glc	17.6	2.7	8.0	9.6	3.2	<LOQ	<LOQ	<LOQ	6.7	0.6	0.6	0.6
ZEN14Sulf	8.8	10.6	24.0	26.1	29.0	31.6	151	220	40.0	4.9	17.1	22.5
ZEN16Glc	23.5	<LOQ	<LOQ	<LOQ	58.1	4.2	7.9	15.1	6.7	2.1	2.8	2.8
α-ZEL	2.9	0.6	0.6	0.6	9.7	1.9	2.2	2.3	13.3	0.6	0.7	0.7
α-ZEL14Glc	23.5	2.9	5.0	5.1	0.0	n.d.	n.d.	n.d.	16.7	3.1	4.2	4.4
β-ZEL	2.9	2.0	2.0	2.0	32.3	3.0	5.1	6.0	10.0	3.5	6.4	6.9
β-ZEL14Glc	2.9	0.7	0.7	0.7	0.0	n.d.	n.d.	n.d.	0.0	n.d.	n.d.	n.d.

*LOD* limit of detection, *LOQ* limit of quantification, *n.d.* not detected

^a^Concentrations below the respective LOQ were assigned a value of LOQ/2; values below LOD were treated as non-contaminated

Apart from the overall high DON levels and a few oats with high NIV concentrations, the prevalence and contamination levels of all other native mycotoxins investigated in this study were well in accordance with the previous survey results from Finland [9]. Oats appear to be the most susceptible commodity, with high mycotoxin levels, especially DON and HT2/T2. Mostly oats, but also some barley and wheat samples from areas 4, 7–10 and 11 (Fig. 2), were contaminated with the highest DON concentrations. In those areas, located in the central and eastern parts of Finland, rainfall occurred during the flowering period of oats, i.e. around the end of June and beginning of July, followed by warm weather, conditions optimal for DON production. Until recently, the dominant DON-producing species in Finland was *Fusarium culmorum* in barley and wheat, but during the past few years, it has been replaced by *Fusarium graminearum*, the populations of which are rapidly spreading not only in Finland but also in other northern countries [37]. As *F. graminearum* is a more prolific DON producer than *F. culmorum*, it may have contributed to the high DON concentrations. In the case of HT2 and T2, the most contaminated samples were from the southern parts of Finland, which had a drier and warmer climate more appropriate for the major HT2/T2 producers in Finnish oats (*Fusarium sporotrichioides* and *Fusarium langsethiae*). NIV is mostly produced by *Fusarium poae* and in smaller amounts by *F. culmorum* and *F. graminearum* in Finland [38]. The generally low concentration levels of NIV in samples with high DON/3Ac-DON contamination support the fact that Finnish *F. culmorum* and *F. graminearum* strains are predominantly of the 3Ac-DON-producing chemotype IA [37]. Finally, ZEN is known to be produced at the end of the growing period before harvesting [39], and the weather at that time was mostly warm and dry in Finland, conditions not favourable for ZEN production, resulting in low to medium levels of contamination.

In this paper, the natural occurrence of some modified mycotoxins (e.g. ZEN16Glc and NIV3Glc) is for the first time presented in some of the commodities analysed here. All of the modified mycotoxins included in the method were quantifiable, with concentrations higher than the respective LOQ of each analyte in at least one sample. DON3Glc (81 %) was by far the most abundant, with the maximum concentrations reaching 6600 μg/kg in a highly DON-contaminated oat sample. Among the other modified mycotoxins, HT2-3-Glc and NIV3Glc were found in 52 and 31 % of the samples, respectively. The modified/native relative proportion, expressed as a molar ratio, was generally in the range of 15–40 % for DON3Glc/DON and slightly higher for HT2-3-Glc/HT2 (30–55 %). The former is in line with previous publications showing a stable relative proportion of DON3Glc to DON at around 20 % [24, 40]. For the latter, no information is currently available in the literature. However, in both cases, barley showed the highest relative proportions of the modified mycotoxins DON3Glc and HT2-3-Glc, and in a few barley samples, the DON3Glc/DON ratio reached levels between 65 and 90 %. Notably, according to our findings, the recently reported ZEN16Glc was detected in more samples than the well-known ZEN14Glc, highlighting the fact that there can still be many modified mycotoxins of significance that may remain unknown. Finally, ZEN14Sulf (29 %) and ZEN16Glc (25 %) were the most prevalent ZEN metabolites overall, while the other ZEN derivatives (ZEN14Glc, α-ZEL, α-ZEL14Glc, β-ZEL and β-ZEL14Glc) were found in less than 15 % of the samples and at low levels.

## Conclusions

This article describes the development and validation of a multi-mycotoxin LC-MS/MS method for the simultaneous analysis of *Fusarium* mycotoxins, including major type A and B trichothecenes and ZEN, as well as certain modified forms in cereals. The method was in-house validated in barley, oats and wheat. Rapid and reliable detection of the target analytes was achieved with this method, offering the ability to obtain quantitative data for modified mycotoxins along with their native forms in cereals during a single run. By utilising this method, we were able for the first time to present occurrence data for a number of modified mycotoxins from cereal grains cultivated in Finland. All of the modified mycotoxins analysed with this method could be detected in the naturally contaminated survey samples, with the most prevalent being DON3Glc, which was also found in the highest concentrations, followed by HT2-3-Glc and NIV3Glc. Regarding native mycotoxins, the contamination levels of DON, NIV and HT2 were the highest, particularly in oats, with their distribution affected by favourable climatic conditions in particular areas for *Fusarium* mycotoxin production.

## Electronic supplementary material

ESM 1 (PDF 24.4 kb)
