# Supplementary material for: Simultaneous determination of major type A and B trichothecenes, zearalenone and certain modified metabolites in Finnish cereal grains with a novel liquid chromatography-tandem mass spectrometric method
Source: Anal Bioanal Chem. 2015 May 3;407(16):4745–55. doi: 10.1007/s00216-015-8676-4 (PMC4446524; doi:10.1007/s00216-015-8676-4)
Supplement: Supplementary file 1 — (PDF 24.4 kb) [file 216_2015_8676_MOESM1_ESM.pdf]

Analytical and Bioanalytical Chemistry

Electronic Supplementary Material

Simultaneous determination of major type A and B trichothecenes, zearalenone and certain modified metabolites in Finnish cereal grains with a novel liquid chromatography-tandem mass spectrometric method

Alexis V. Nathanail, Jenna Syvähuoko, Alexandra Malachová, Marika Jestoi, Elisabeth Varga, Herbert Michlmayr, Gerhard Adam, Elina Sieviläinen, Franz Berthiller, Kimmo Peltonen

Table S1 Method performance characteristics and validation parameters determined in barley

Analyte	SSE (%)	LOD (μg/kg)	LOQ (μg/kg)	R^2	Ion ratio (Quan/Qual)	Apparent recovery ± RSD_R (%) (n = 18)		
						Low	Medium	High
3Ac-DON	109	3.4	10.2	0.993	0.3	110 ± 19	97 ± 9	99 ± 6
DON	78	1.2	3.6	0.995	1.4	102 ± 15	99 ± 12	90 ± 7
DON3Glc	74	2.8	8.4	0.994	1.1	93 ± 18	103 ± 27	101 ± 3
HT2	116	3.1	9.3	0.999	1.1	98 ± 6	102 ± 5	102 ± 6
HT2-3-Glc	121	4.1	12.3	0.993	0.6	100 ± 22	94 ± 26	102 ± 9
NIV	81	3.1	9.3	0.995	1.3	95 ± 15	99 ± 10	100 ± 9
NIV3Glc	59	3.5	10.5	0.989	4.9	90 ± 35	92 ± 23	96 ± 29
T2	107	2.5	7.5	0.999	0.8	96 ± 6	103 ± 4	103 ± 4
ZEN	84	2.0	6.0	0.999	1.2	115 ± 15	115 ± 5	116 ± 4
ZEN14Glc	89	0.1	0.3	0.998	2.4	93 ± 9	98 ± 26	99 ± 4
ZEN14Sulf	100	0.1	0.3	0.999	7.6	98 ± 8	102 ± 4	102 ± 4
ZEN16Glc	88	1.0	3.0	0.992	2.8	96 ± 29	97 ± 16	98 ± 8
α-ZEL	73	0.1	0.3	0.998	1.1	98 ± 10	99 ± 5	102 ± 4
α-ZEL14Glc	86	0.8	2.4	0.996	2.3	103 ± 18	103 ± 11	103 ± 5
β-ZEL	73	0.2	0.6	0.998	1.2	106 ± 12	98 ± 7	100 ± 8
β-ZEL14Glc	93	0.2	0.6	0.999	8.7	102 ± 7	102 ± 6	103 ± 4

Notes: SSE, signal suppression/enhancement ratio; LOD, limit of detection; LOQ, limit of quantification; R^2, coefficient of determination; Quan, quantifier ion; Qual, qualifier ion; RSD_R, inter-day precision

Table S2 Method performance characteristics and validation parameters determined in oats

Analyte	SSE (%)	LOD (µg/kg)	LOQ (µg/kg)	R^2	Ion ratio (Quan/Qual)	Apparent recovery ± RSD_R (%) (n = 18)		
						Low	Medium	High
3Ac-DON	122	5.1	15.3	0.988	0.3	97 ± 29	92 ± 15	98 ± 7
DON	77	1.8	5.4	0.996	2.7	91 ± 11	98 ± 10	100 ± 11
DON3Glc	114	3.9	11.7	0.989	1.1	96 ± 13	99 ± 7	94 ± 13
HT2	75	2.9	8.7	0.997	1.0	96 ± 16	100 ± 20	101 ± 8
HT2-3-Glc	62	4.2	12.6	0.986	0.6	101 ± 29	84 ± 7	106 ± 17
NIV	85	3.4	10.2	0.982	1.4	106 ± 18	111 ± 15	100 ± 15
NIV3Glc	93	5.3	15.9	0.989	4.5	114 ± 28	93 ± 10	87 ± 20
T2	101	2.6	7.8	0.999	1.0	98 ± 11	100 ± 27	100 ± 7
ZEN	79	3.1	9.3	0.999	1.3	92 ± 13	92 ± 17	96 ± 10
ZEN14Glc	53	0.2	0.6	0.995	4.0	111 ± 16	104 ± 9	106 ± 12
ZEN14Sulf	116	0.1	0.3	0.998	6.9	103 ± 8	101 ± 12	101 ± 9
ZEN16Glc	75	0.3	0.9	0.989	4.4	108 ± 24	102 ± 12	106 ± 9
α-ZEL	69	0.3	0.9	0.997	1.1	92 ± 14	92 ± 18	97 ± 9
α-ZEL14Glc	110	2.5	7.5	0.990	2.5	115 ± 21	99 ± 13	101 ± 11
β-ZEL	92	0.4	1.2	0.997	1.2	100 ± 15	94 ± 22	97 ± 8
β-ZEL14Glc	105	5.1	15.3	0.995	9.1	97 ± 29	92 ± 15	98 ± 7

Notes: SSE, signal suppression/enhancement ratio; LOD, limit of detection; LOQ, limit of quantification; R^2, coefficient of determination; Quan, quantifier ion; Qual, qualifier ion; RSD_R, inter-day precision
